# Melatonin-Mediated Nrf2 Activation as a Potential Therapeutic Strategy in Mutation-Driven Neurodegenerative Diseases

**DOI:** 10.3390/antiox14101190

**Published:** 2025-09-28

**Authors:** Lucía Íñigo-Catalina, María Ortiz-Cabello, Elisa Navarro, Noemí Esteras, Lisa Rancan, Sergio D. Paredes

**Affiliations:** 1 Department of Biochemistry and Molecular Biology, School of Medicine, Complutense University of Madrid, 28040 Madrid, Spain; luinigo@ucm.es (L.Í.-C.); elisnava@ucm.es (E.N.); nesteras@ucm.es (N.E.); lisaranc@ucm.es (L.R.); 2 Department of Physiology, School of Medicine, Complutense University of Madrid, 28040 Madrid, Spain; marort09@ucm.es; 3 Neurochemistry Research Institute, Complutense University of Madrid, 28040 Madrid, Spain; 4 CIBERNED (Network Center for Biomedical Research in Neurodegenerative Diseases), 28029 Madrid, Spain; 5 Instituto Ramón y Cajal de Investigación Sanitaria (IRYCIS), 28034 Madrid, Spain; 6 Group of Neurodegenerative Diseases, Hospital Universitario 12 de Octubre Research Institute (imas12), 28041 Madrid, Spain

**Keywords:** melatonin, Nrf2, familial neurodegenerative diseases, tauopathies, Parkinson’s disease, neurodegeneration, mutations, oxidative stress, neuroinflammation

## Abstract

Neurodegeneration is intrinsically linked to aging through processes such as oxidative stress, mitochondrial dysfunction, and chronic inflammation. Nuclear factor erythroid 2-related factor 2 (Nrf2) emerges as a central transcription factor regulating these molecular events and promoting cytoprotective responses. In neurodegenerative diseases, notably, frontotemporal dementia (FTD) and Parkinson’s disease (PD), genetic mutations—including *MAPT*, *LRRK2*, *PINK1*, *PRKN*, and *SNCA*—have been reported to alter Nrf2 signaling, both in vitro and in vivo. Melatonin, a neurohormone widely known for its strong antioxidant and mitochondria-stabilizing properties, has been shown to activate Nrf2 and restore redox balance in several experimental models of neurodegeneration. Its effects include a reduction in tau hyperphosphorylation, α-synuclein aggregation, and neuroinflammation. While most data are derived from sporadic models of Alzheimer’s disease and PD, emerging evidence supports a role for melatonin in familial forms of FTD and PD as well. Thus, targeting Nrf2 through melatonin may offer a promising approach to mitigating neurodegeneration, especially in the context of mutation-driven pathologies. Further investigation is warranted to explore mutation-specific responses and optimize the therapeutic strategies.

## 1. Introduction

### 1.1. Aging and Neurodegenerative Diseases (NDs)

Due to the lower prevalence of infectious and acute diseases, along with the chronification of many conditions—such as neoplastic diseases—that previously shortened human life expectancy, population aging rates are increasing. It is known that aging is a common risk factor in many pathologies without a known cure, including neurodegenerative diseases (NDs) or cancer. Therefore, it cannot be defined simply as an increase in lifespan, as it is associated with phenotypic changes at multiple levels of the organism. Understanding the biology of the aging process can help modulate the factors involved and contribute not only to an increasing life expectancy, but also to improving the healthspan [1,2].

Aging is widely described as a multifactorial process involving interconnected mechanisms that drive progressive cellular dysfunction. It is a state described no longer by the nine widely known distinctive hallmarks [3], but has recently been updated to twelve: genomic instability, telomere attrition, epigenetic alterations, the loss of proteostasis, deactivated macroautophagy, the dysregulation of nutrient sensing, mitochondrial dysfunction, cellular senescence, stem cell depletion, altered intercellular communication, chronic inflammation, and dysbiosis. These hallmarks are not isolated cellular processes; they are interconnected and affect most structural and functional features of the cell, especially those related to the homeostatic imbalance and deficient response to oxidative stress [4]. Underlying these particular processes, inflammation and increased oxidative stress are common pre-steps to many of them. Reactive oxygen and nitrogen species (ROS and RNS, respectively) exist under conditions of homeostasis; however, an imbalance between their generation and neutralization leads to changes in the physiological concentrations and the progression towards oxidative stress, which results not only in aging but also in various pathological conditions, often including mitochondrial dysfunction, inflammation, DNA damage, and NDs [5].

Our brain cells—especially neurons, but also other cell types—are particularly vulnerable to disruptions in mitochondrial function and redox imbalance, due to their high energy demands and low regenerative capacity. This vulnerability lies at the core of the mitochondrial free radical theory of aging, which posits that aging exacerbates this imbalance by increasing mitochondrial DNA (mtDNA) mutations and impairing antioxidant defenses, particularly in vulnerable brain regions like the hippocampus and substantia nigra [6,7]. This increased susceptibility with age contributes to the pathogenesis of NDs [8,9,10].

NDs are a heterogeneous group of pathologies that involve the loss of neurons in either the central nervous system or the peripheral nervous system. This degeneration of neural circuits leads to cognitive decline, altering memory, behavior, and/or movement. Therefore, these chronic age-related diseases are becoming increasingly significant for public health.

Although much is yet to be explored about the pathological mechanisms underlying neurodegeneration, research over the recent decades has shed light on NDs’ hallmarks [11], which are closely associated with the well-known hallmarks of aging [3]. Maintained neuroinflammation, increased oxidative stress, protein aggregation, and mitochondrial dysfunction are some of the shared pathological events underlying this heterogeneous group of neurological disorders [12,13], and have been specifically described in Alzheimer’s disease (AD) and Parkinson’s disease (PD).

Currently, there is still no effective medical treatment for most of these conditions, as the therapies are mainly symptomatic rather than disease-modifying. This, along with the lack of early diagnostic biomarkers, highlights the need for further investigation in this field. Although genetic variation appears to play a key role in the context of aging [14], a complex interaction between both genetic and environmental factors is determinant in the onset and progression of sporadic forms of NDs, which are the most prevalent [12,15].

From a translational point of view, there is growing interest in compounds that have shown efficacy in delaying aging and its associated pathologies in animal models, although the step to the clinic is still difficult to take. They are known as geroprotectants and can act by preventing oxidation, regulating proteostasis and mitochondrial function [16]. In this scenario, where multiple natural exogenous molecules are emerging as possible dietary supplements [17], melatonin may also be considered as an interesting option for research. Melatonin, a neurohormone best known for regulating circadian rhythms, has been increasingly studied in recent years for its antioxidant, anti-apoptotic, and neuroprotective properties. These effects are largely attributed to its ability to activate the nuclear factor erythroid 2-related factor 2 (Nrf2), encoded by the *NFE2L2* gene, pathway [18].

### 1.2. Nrf2 and NDs

Given the established hallmarks of NDs [11] and recognizing that Nrf2 induces the expression of cytoprotective genes and inhibits the activation of pro-inflammatory genes, this transcription factor has been suggested as a potential target against NDs [12,13,19]. Indeed, oxidative stress and neuroinflammation are two key neurodegenerative features that are addressed by intrinsic Nrf2-dependent detoxification mechanisms.

Nrf2 is considered a master antioxidant regulator. Under healthy conditions, it is constitutively translated but blocked in the cytosol by KEAP1, which facilitates its proteasomal degradation via the Cul3-Rbx1 ubiquitin ligase complex. When KEAP1 becomes saturated, newly synthesized Nrf2 remains unbound and translocates to the nucleus, where it dimerizes with sMAF proteins and binds to antioxidant response elements (AREs) or electrophile response elements (EpREs) present in the promoter region of specific genes. In this activated state, Nrf2 induces the expression of antioxidant as well as cytoprotective genes such as heme-oxygenase-1 (HO-1), NAD (P)H:quinone oxidoreductase 1 (NQO1), or glutamate cysteine ligase catalytic subunit (GCLC) [20].

The Nrf2 signaling pathway might be induced by several factors that disrupt its interaction with KEAP1. These inducers typically trigger cellular stress responses, particularly oxidative or electrophilic stress [21]. It has been widely described that Nrf2 activation occurs mainly through the modification of cysteine residues in KEAP1 (a redox sensor) by these cellular-stress-related molecules. To a lesser degree, Nrf2 release can be due to binding competition, especially by p62 interfering with KEAP1. In addition, the kinases PI3K and AKT play an important role in the status of this factor, as they phosphorylate, and, thus, inhibit, GSK-3β (Nrf2’s inhibitor), which makes them indirect activators of Nrf2 [22]. Nrf2 modulation has, therefore, attracted pharmacological interest, and a couple of therapeutic inducers have already been tested and approved in the context of NDs, such as dimethyl fumarate (DMF) for multiple sclerosis [23] and omaveloxolone for Friedrich’s ataxia [24].

Beyond its antioxidant role, Nrf2 is a key anti-inflammatory mediator, due to its ability to inhibit the classical pro-inflammatory transcription factor NF-κB by inducing its previously mentioned antioxidant target genes, hence inhibiting the expression of pro-inflammatory cytokines and enzymes [20].

When it comes to NDs, the Nrf2 signaling pathway is known to be altered, and different variations in its components have been reported. For instance, postmortem brains of patients, despite having lower nuclear levels of Nrf2, show higher levels of glial HO-1 and NQO1 [25]. Moreover, animal models of AD have reported an attenuation of the Nrf2 signaling cascade [26]. Specifically, the downstream antioxidant gene expression (NQO1, glutathione synthetic enzymes) is reduced in these models, a condition further aggravated by the overactivity of GSK-3β [26]. On the contrary, the Nrf2 accumulation and activation of NQO1 and HO-1 have been observed both in PD patients [27,28] and animal models [29,30], probably due to the higher levels of oxidative stress. However, the successive levels of phase II enzymes are insufficient to protect neurons, which could indicate the dysregulation of this pathway [25]. Furthermore, the disruption of the respiratory chain, decreased mitochondrial Ca^2+^ trafficking, and increased ROS production have been described in a transgenic mice model of amyotrophic lateral sclerosis [31], a disease in which Nrf2 activity is thought to be downregulated, especially when it is associated with mutations in the copper–zinc superoxide dismutase type 1 (SOD1) protein gene [12].

Nrf2 not only is essential in regulating oxidative stress and neuroinflammation, but it is also implicated in proteostasis maintenance by modulating the proteasome subunit expression and upregulating essential genes for the ubiquitin-proteasome system, the main proteolytic system controlling autophagy. These two processes are key for the clearance of misfolded or damaged proteins prone to aggregation—a pathological hallmark in NDs. In this context, it has been reported that Nrf2 improves neuronal survival in PD by decreasing α-synuclein levels [32].

In addition, Nrf2 also exerts cytoprotective effects by regulating mitochondrial homeostasis. First, it plays an important role in energy production, based on maintaining the mitochondrial membrane potential and increasing the availability of substrates and enzymes for the respiratory chain and ATP synthesis [33]. In this regard, proper Nrf2 function is critical for preventing bioenergetic impairments and excessive mitochondrial ROS production [34]. In addition, Nrf2 activation prevents the oxidative-stress-induced opening of the mitochondrial permeability transition pore (mPTP), thus preventing mitochondrial Ca^2+^ overload in neurons where the efflux is compromised—as in PD or tauopathies [35]. Moreover, Nrf2 promotes mitochondrial biogenesis by regulating the expression of various genes and interacting with the transcriptional coactivator peroxisome proliferator-activated receptor (PPAR) γ coactivator 1-alpha (PGC-1α) [36]. PGC-1α is considered a master regulator of mitochondrial biogenesis, as it activates key transcription factors for mitochondrial function such as PPARs, Estrogen-Related Receptors (ERRs), and Nuclear Respiratory Factors (NRF1 and NRF2) [36]. Moreover, Nrf2 has been reported to be directly related to autophagy and, therefore, mitophagy, as it takes part in the regulation of autophagosomal degradation mediators like p62 [37]. It has been proven that Nrf2 enhances mitochondrial dynamics and integrity, and it is also thought to participate in the transport and motility of this organelle [20,32,34,38].

In NDs, such as AD, frontotemporal dementia (FTD), and PD, a common hallmark is the dysregulation of protein homeostasis, mitochondrial function, and Ca^2+^ signaling [12,13,39]. In tauopathies like AD and FTD, the accumulation of misfolded proteins—such as tau and β-amyloid—contributes to increased oxidative stress, the disruption of Ca^2+^ homeostasis, and mitochondrial dysfunction [40]. Similarly, in PD, impairments in the mitochondrial respiratory chain have been associated with defective mitophagy, altered mitochondrial Ca^2+^ regulation, and elevated oxidative stress, and accumulated α-synuclein [41,42]. Taken together, these findings suggest that the activation of the Nrf2 may represent a potential therapeutic strategy for addressing pathological hallmarks of NDs.

### 1.3. Melatonin and Nrf2

Melatonin (N-acetyl-5-methoxytryptamine) is a hormone produced by the pineal gland in the brain. Its production is closely regulated by exposure to light, making it a key factor in the maintenance of circadian rhythms. Its levels begin to rise in the evening, peak during the night, and decrease in the early morning, thus regulating sleep–wake cycles under the control of the suprachiasmatic nucleus in the hypothalamus [43]. In addition to its role in circadian regulation, since its discovery in 1958 [44,45], melatonin has attracted significant scientific interest due to its role in various physiological processes, especially in the brain, as it can cross and bind to the blood–brain barrier, thereby performing various functions within it [46]. Increasing evidence emphasizes the physiological significance of extrapineal melatonin, since it is also synthesized locally in various human tissues by two key enzymes: arylalkylamine N-acetyltransferase (AANAT) and acetylserotonin O-methyltransferase (ASMT) [47,48]. It is considered to have reparative effects as an anti-inflammatory, an antioxidant, a regulator of apoptosis and autophagy, or, on a larger scale, an anticarcinogenic agent [49,50].

With respect to its antioxidant function, melatonin’s activity extends well beyond its classical characterization as a systemic free-radical scavenger. It includes both the direct neutralization of ROS/RNS and the indirect, receptor-mediated upregulation of endogenous antioxidant defenses [51]. Numerous experimental studies describe the indirect, receptor-mediated mechanisms by which melatonin enhances the cellular antioxidant capacity. Canonical melatonin receptors (MT_1_/MT_2_) have been shown to trigger intracellular signaling, leading to the upregulation of antioxidant enzymes, including superoxide dismutases, catalase, and glutathione peroxidase, predominantly via the Nrf2-ARE pathway [52,53,54]. Some years ago, it was established that melatonin plays a pivotal role in promoting Nrf2 stability and nuclear translocation. This effect seems to be achieved by shielding Nrf2 from proteasomal degradation, thereby promoting its activation and nuclear translocation [55].

Furthermore, more recent work indicates a functional interaction between melatonin and mitochondrial sirtuin 3 (SIRT3), which results in the activation of mitochondrial antioxidant defenses and improved redox homeostasis [56]. In addition, and in relation to one of the most important organelles in the redox response, melatonin’s actions regulate mitochondrial dynamics, including processes such as mitophagy and biogenesis [57]. This regulation occurs due to melatonin’s accumulation in mitochondria, which is facilitated both by the transporter-mediated uptake and by local synthesis through mitochondrial isoforms of AANAT and ASMT enzymes [47,48]. The functional relevance of these mechanisms is highlighted by studies in both in vitro and in vivo models of brain oxidative stress, neurodegeneration, and aging. In these contexts, melatonin and its metabolites confer neuroprotection against excitotoxins, ischemia/reperfusion, neurotoxins (rotenone), and chemotherapeutic agents [58,59,60]. Conversely, other authors have recently revealed how, in some models of models of tauopathy, antioxidant effects and neuroprotection appear to be dissociated [61], making the need for further research in this field more evident.

Additionally, melatonin effectiveness has been tested in patients with NDs, although there is no clinical evidence directly relevant to the scope of the present review. A six-month, randomized, placebo-controlled, multicenter trial showed that add-on prolonged-release melatonin produced positive effects on cognitive functioning and sleep maintenance in AD patients compared with placebo [62]. Moreover, studies concerning melatonin’s possible effects on AD progression (NCT04522960) and on sleep problems associated with AD (NCT00940589) and PD (NCT02768077, NCT02789592, NCT05307770, and NCT03258294) have been carried out.

When considering melatonin as a possible therapeutic strategy, some limitations must be considered [63]. These include the poor bioavailability of oral melatonin [64], inconsistent and unpredictable dosing [65], and highly variable pharmacokinetic parameters among individuals [63,66]. Moreover, the intrinsic characteristics of the molecule and its receptor polymorphisms must also be considered, as its efficacy may be inconsistent at oral therapeutic doses despite its lipophilic nature [67], and the human response to melatonin is strongly influenced by the timing of the administration [63]. In addition, regarding melatonin’s activity, chronically activating Nrf2 may favor tumor progression [68], impaired glucose metabolism, reduced tissue regeneration, and abnormal heart tissue remodeling [69].

Nevertheless, taken together, the induction of the Nrf2 pathway by means of melatonin—a clear trigger of its downstream anti-inflammatory and antioxidant mechanisms—represents a promising strategy for addressing NDs. Indeed, this route is of particular interest in some familial NDs, where a further relationship between the mutated gene/s and Nrf2 has been identified.

## 2. Familial Tauopathies and Nrf2/Melatonin Axis

Tauopathies represent a broad spectrum of phenotypically diverse NDs characterized by the accumulation of misfolded tau protein in brain aggregates. They include both dementias like AD—where tau forms hyperphosphorylated proteinaceous inclusions termed neurofibrillary tangles (NFTs)—and movement disorders such as progressive supranuclear palsy, corticobasal degeneration, or FTD with parkinsonism linked to chromosome 17 (FTDP-17) [70].

Tau protein is a microtubule (MT)-associated intracellular protein whose main physiological function is to assemble and stabilize microtubules, maintaining the neuronal cytoskeleton and axonal transport. It is encoded by the microtubule-associated protein tau (*MAPT)* gene, located in chromosome 17. The alternative splicing of this gene generates six different tau isoforms in the human brain. These isoforms differ in the presence or absence of exons 2, 3, and 10 in the mature resulting protein. Specifically, the alternative splicing of exons 2 and 3 leads to isoforms with 0, 1, or 2 N-terminal repeats (0N, 1N, and 2N), while the alternative splicing of exon 10 results in mature tau with either three (3R) or four (4R) MT-binding repeats in the MT-binding domain. Isoforms 3R and 4R are equally expressed in the adult brain, and altering this ratio is enough to trigger neurodegeneration, as was demonstrated in some forms of FTD with parkinsonism linked to chromosome 17 (FTDP-17) for the first time [70].

One of the primary mechanisms that disrupts this 3R/4R equilibrium involves mutations in the *MAPT* gene, particularly those affecting exon 10 splicing. These mutations often promote the inclusion of exon 10 in the mature transcript, resulting in an overrepresentation of 4R tau relative to 3R tau. Other *MAPT* mutations impair the functional properties of tau directly—either by reducing its affinity for microtubule binding or by increasing its propensity to aggregate in its unbound form. Both mechanisms are closely interconnected. Moreover, mutations affecting *MAPT* alternative splicing may also reproduce these effects, as the various isoforms exhibit different microtubule-binding affinities [70,71].

Hence, *MAPT* mutations are considered to be sufficient to trigger neurodegeneration. They are deemed as a direct cause of familial FTD, which is a relatively common and heterogeneous neurodegenerative disorder [70,72]. AD also warrants attention in this context. As one of the most prevalent NDs, together with PD or amyotrophic lateral sclerosis, AD is genetically driven in approximately 1–5% of cases [13,15]. These familial cases have mainly been correlated to mutations in three key proteins: amyloid precursor protein (*APP*), presenilin 1 (*PSEN1*), and presenilin 2 (*PSEN2*). Furthermore, specific polymorphisms of certain genes like *APOE*, *TREM2*, and *PLCG2* have been linked to increased susceptibility to sporadic AD. Nevertheless, abnormal tau forms have also been associated with the development of this disease [70].

As previously mentioned, Nrf2 represents a potential target in ND as a result of its antioxidant and cytoprotective properties. In the context of familial tauopathies, a relationship between the *MAPT* gene expression and Nrf2 downstream pathway has been suggested. To begin with, NRF2/sMAF binding has been observed at a functional ARE (Antioxidant Response Element) SNP (Single Nucleotide Polymorphism) in *MAPT* intron 1. Specifically, this SNP is related to a protective allele for common tauopathies such as PSP, PD, and CBD. Hence, the previously mentioned binding suggests that Nrf2 could exert the same positive role for these tauopathies [73]. In addition, an inverse correlation between Nrf2 and tau gene expression has been described. In experimental models where the *MAPT* gene was inhibited as a potential treatment for traumatic brain injury, researchers observed antioxidant and anti-apoptotic effects—suggesting that these outcomes were mediated via the activation of the Nrf2/HO-1 signaling pathway [74]. Moreover, the association between Nrf2 and tau gene expression has also been established in the opposite direction, as Nrf2-knockout mice models show increased levels of tau [75,76].

Notably, this inverse regulation persists even in the presence of pathogenic *MAPT* mutations. A study investigating Nrf2’s role in macroautophagy reported a close interplay in expression: Nrf2-deficient mice co-expressing *APP* and *MAPT* mutations—specifically Hs*APP*V717I and Hs*MAPT*P301L—showed increased intracellular tau aggregates as well as reduced autophagy [75]. In addition, neuroinflammation, the dysregulation of Ca^2+^ homeostasis, and tau hyperphosphorylation in an AAV-hTAUP301L mouse model were shown to be mitigated by treatment with the Nrf2 inducer dimethyl fumarate (DMF) [77].

In this context, Nrf2 appears to play a key role in supporting overall brain health. Authors evaluating cognitive decline in mice carrying the P301S mutation in *MAPT*, both in the presence and absence of Nrf2, reported that Nrf2 helps preserve brain function [78]. Moreover, another study revealed that impaired and downregulated Nrf2 signaling in the brains of the same model may contribute to neuroinflammation and tauopathy [79]. The triggering of synaptic toxicity and the inhibition of Nrf2 transcriptional activity due to KEAP1 acetylation were suggested as possible mechanisms in this P301S-mutated tau model [80]. Additionally, pharmacological strategies aimed at restoring Nrf2 activity have yielded promising results. For instance, benfotiamine treatment has proven effective in exerting neuroprotective effects by activating the Nrf2/ARE pathway in a P301S mouse model of tauopathy, representing a promising therapeutic approach for diseases with tau pathology, such as AD, FTD, and progressive supranuclear palsy [81].

Besides neuronal function being affected by the impairment of Nrf2 signaling under *MAPT* mutations, this relationship is also seen in other cell types. A study concerning the connection between chemokines released by neurons overexpressing tau P301L and the Nrf2 system shows that the CX3CR1/Nrf2 axis plays an essential role in microglial function [82]. Moreover, astrocytes from *MAPT* P301S mice have been reported to acquire harmful signatures in response to tau pathology, involving inflammatory and stress-activated cytoprotective Nrf2 pathways [83].

It is important to consider that all the above-mentioned investigations explore the interaction between Nrf2 and *MAPT* mutations specifically affecting exon 10, notably P301S and P301L, both of which alter the biochemical properties of the 4R tau isoform [71]. These variants affect tau protein functionality directly. However, the mechanism by which other *MAPT* mutations may affect the Nrf2 transcriptional signaling pathway has not been explored in depth yet. Considering that different mutations in the *MAPT* gene can lead to diverse molecular, functional, and clinical outcomes, further investigation is clearly warranted [70,71,84].

Focusing on FTD models, a more specific framework among familial tauopathies, the literature about the Nrf2/ARE molecular cascade is limited due to the heterogeneous nature of the disease. Nevertheless, the beneficial effects of Nrf2 activation appear to be maintained in this context as well. It was reported that, in a *MAPT* P301S mouse model of FTD, the overexpression of mutant tau induced mitochondrial and synaptic dysfunction as well as stress responses [85]. These effects were recovered when treating mice with hydromethylthionine in a mechanism mediated by the Nrf2 pathway upregulation, among other mechanisms described by the authors [85].

**Figure 1:**
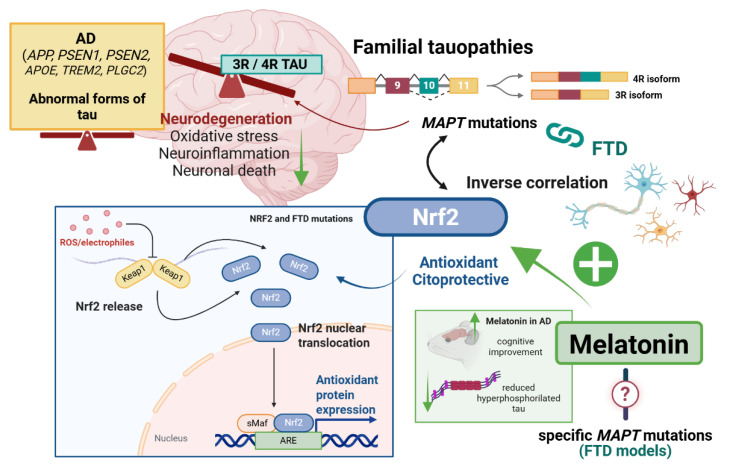
**FTD-related *MAPT* mutations and the potential impact of melatonin as an inducer of Nrf2. Top left**: AD has been linked to mutations in *APP*, *PSEN1*, *PSEN2*, *APOE*, *TREM2*, and *PLGC2* genes, as well as abnormal forms of tau which maintain the 3R/4R isoform balance. **Top right**: Specific *MAPT* mutations, which are known to dysregulate the 3R/4R isoforms equilibrium, are directly associated with FTD. **Bottom left**: *MAPT* gene mutations present an inverse correlation with the Nrf2 pathway, whose molecular mechanisms remain unknown. **Bottom right**: Melatonin is already known to trigger beneficial effects in AD models through Nrf2 activation, and a similar protective role is suggested for familial FTD models. *Created with BioRender 201*.

In the context of familial AD, the balance between 3R and 4R isoforms remains steady [70]. Nevertheless, *MAPT* mutations can cause either 3R/4R-predominant tauopathies or tauopathies with an equal expression of both isoforms. Keeping this in mind, both transgenic models presenting alterations in *APP*, *PSEN1*, *PSEN2*, or specific *MAPT* mutations (such as P301S) may be employed in the study of the Nrf2 pathway in this pathology (Figure 1).

In this framework, Nrf2’s cascade activation has been reported to be beneficial both in animal models and patients (Figure 1). Recent research shows that the loss of Nrf2 in tau-based P301S mice results in accelerated cognitive decline, suggesting that Nrf2 protects brain function in a model of AD [78]. Furthermore, a natural compound termed antroquinonol has been stated to upregulate Nrf2 in the brains of APP/PS1 [86] and 3TgXAD (PS1M146V, APPSwe, and tauP301L) [87] AD animal models, improving cognitive impairment.

Regarding AD patients, it has been shown that Nrf2 reduces phosphorylated tau levels by inducing the expression of the autophagy adaptor protein NDP52 [76]. In brain cortical samples, a clear NDP52-tau association was observed, along with an inverse correlation between phosphorylated tau levels and NDP52 expression [76,88]. Furthermore, studies analyzing Nrf2’s role in macroautophagy regulation have shown that neurons from AD patients exhibiting high levels of APP or MAPT also express elevated levels of Nrf2. This upregulation is interpreted as a compensatory cellular response aimed at degrading accumulating protein aggregates through the autophagic machinery [75]. Consequently, these findings support the notion that targeting Nrf2 to enhance autophagy could be a promising therapeutic strategy in tauopathies. In line with this hypothesis, sulforaphane, a potent Nrf2 activator known for upregulating detoxification, antioxidant, and immune-modulating enzymes, has been shown to inhibit tau phosphorylation [88], further reinforcing Nrf2’s potential in mitigating tau-driven neurodegeneration.

Considering the growing body of preclinical and clinical evidence, it is noteworthy that multiple clinical trials are currently underway to evaluate the efficacy of small-molecule Nrf2 activators in AD patients [32]. The early findings regarding these drugs are quite encouraging [89].

Consistent with these facts is the notion that melatonin, previously discussed as one of the activators of the Nrf2 transcriptional pathway, may serve as an effective treatment for tauopathies (Figure 1). Several studies indicate that melatonin exerts neuroprotective effects in tauopathy models. However, its benefits via Nrf2 activation are mostly established in AD pathology, as, to our knowledge, there is no published data yet about how melatonin modulates Nrf2 in FTD models [90,91].

In this context, melatonin has been found to reduce tau hyperphosphorylation, oxidative stress, and neuronal failure in vitro. The authors reported that melatonin was able to reduce inflammation, tau hyperphosphorylation by GSK-3β, and oxidative stress in Neuro2A cells [92] (Figure 1). Moreover, melatonin was shown to improve mitochondrial biogenesis, structure, and function by activating Nrf2, among other pathways, in a HEK293-APPSwe cell model of AD [57]. Additionally, melatonin treatment in SH-SY5Y cells and primary neurons induced Nrf2 nuclear translocation, offering protection against Aβ-mediated neuronal dysfunction [93].

In vivo evidence further supports the therapeutic promise of melatonin. In animal models of AD, melatonin administration has been associated with cognitive improvement via Nrf2 activation (Figure 1). For example, melatonin has been reported to prevent memory dysfunction by attenuating oxidative stress in a high-fat-diet-induced AD model [94]. In addition, treating a streptozotocin-induced rat model of sporadic AD with melatonin improved cognitive performance and reduced the markers of neuroinflammation [95]. Furthermore, other authors suggest that melatonin-alkylbenzylamine hybrids may induce Nrf2 and the expression of its downstream proteins, proving effective for treating AD [96].

However, as mentioned before, the efficacy of melatonin in treating familial tauopathies linked to mutations in the *MAPT* gene via the activation of the Nrf2/ARE molecular cascade remains unexplored (Figure 1). In view of the fact that this molecule has already been linked to a reduction in hyperphosphorylated tau fibrils [97,98] and that its exogenous form can improve memory impairment and reduce AD-like hyperphosphorylation through its antioxidant function [99], melatonin as an inducer of the Nrf2 pathway should be further investigated as a potential treatment for familial tauopathies.

## 3. PD and Nrf2/Melatonin Axis

PD is a progressive neurodegenerative disorder characterized by the altered proteostasis of α-synuclein in dopaminergic neurons, which results in cytoplasmic aggregates defined as Lewy bodies. These two main pathophysiological events occur together with some of the previously mentioned hallmarks of neurodegeneration, such as oxidative stress, mitochondrial dysfunction, and neuroinflammation [15,100]. Together, these processes drive the loss of dopaminergic neurons, mainly in the substantia nigra, and, consequently, in the striatum.

Genetic mutations in key PD-linked genes, such as *PRKN* (Parkin), *PINK1* (PINK1), *LRRK2* (LRRK2), *PARK7* (DJ-1), and *SNCA* (α-synuclein), contribute to familial forms of the disease and may disrupt the cell’s antioxidant responses [15]. This occurs due to the crucial roles that proteins encoded by the mentioned genes play in the development of this pathology. Both Parkin and PINK1 proteins play a central role in mitochondrial quality control; mutations in either gene result in the accumulation of dysfunctional mitochondria, thereby increasing oxidative stress and triggering neuronal cell death [101,102]. LRRK2 is a key enzyme that acts as a signaling scaffold, participating in multiple cellular signaling processes. Mutations in it trigger neurodegeneration by diverse molecular mechanisms [103]. DJ-1’s main function is to protect cells against oxidative damage and cell death, so its loss generally renders dopaminergic neurons more vulnerable to degeneration [104]. Lastly, α-synuclein, whose main role is linked to synaptic vesicle trafficking, aggregates into Lewy bodies due to mutations in the *SNCA* gene [105].

While Nrf2 has long been proposed to confer neuroprotection in PD, the direct impact of PD-related mutations on Nrf2 function remains incompletely characterized, particularly in terms of direct mechanistic disruptions, such as altered protein expression, impaired nuclear translocation, or transcriptional repression.

**Figure 2:**
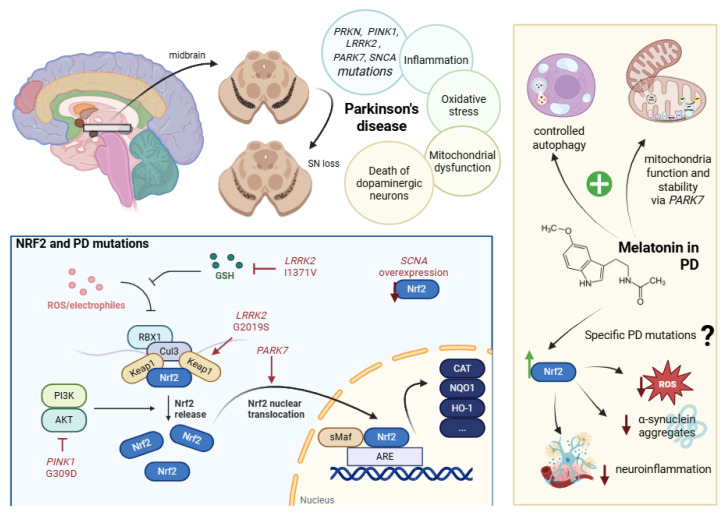
**PD-related mutations and the possible impact of melatonin. Top left**: PD is characterized by the loss of dopaminergic neurons in the substantia nigra of the midbrain, driven by several synergistic contributing processes, among which PD-related gene mutation can be implicated. **Bottom left**: Molecular regulation of Nrf2 by these mutations and how they affect its different activation and translocation states. **Right**: Suggested protective role of melatonin in PD via increased cellular dynamics stability and Nrf2 activation. *Created with BioRender 201*.

The *SNCA* gene product, α-synuclein, is also implicated in the dysregulation of the Nrf2 pathway. The overexpression of α-synuclein has been shown to suppress Nrf2 protein levels (Figure 2) and promote ferroptosis in mouse models, establishing a feedback loop between oxidative stress and neurotoxicity [106]. Furthermore, mutant α-synuclein interferes with Nrf2 transcriptional activity at specific genomic loci, such as the microtubule-associated gene *Map1b*, contributing to axonal dysfunction [107]. More broadly, Nrf2 deficiency has been found to exacerbate neurodegeneration in the presence of α-synuclein overexpression, with worsened behavioral deficits observed in knockout mouse models [108]. Additionally, proximity proteomics identified ZNF746/PARIS, an established PD-linked protein, as a direct repressor of Nrf2 transcriptional activity, linking the transcriptional suppression of Nrf2 to increased neuronal oxidative stress and apoptosis [109].

Emerging evidence has begun to directly interrogate how PD-associated mutations affect Nrf2 expression and activity (Figure 2). As an example, the *PINK1* G309D mutation was shown to inhibit Nrf2 nuclear translocation and downstream antioxidant expression by disrupting p38 and Akt phosphorylation (Figure 2), thereby impairing Nrf2 pathway activation under proteasomal stress [110]. Reciprocally, Nrf2 was found to transcriptionally regulate *PINK1* expression under oxidative conditions, illustrating a feedback axis between mitochondrial function and Nrf2 pathway activity [111]. *LRRK2*—G2019S is the most common mutation in both familial and sporadic PD, and it is associated with an increase in LRRK2 kinase activity. In line with other *LRRK2* mutations, G2019S suppresses Nrf2 expression and its transcriptional activity (Figure 2) in both mouse and cell models, potentially through KEAP1 upregulation and the subsequent Nrf2 degradation, attenuating the antioxidant response [112,113]. In line with this fact, another *LRRK2* mutation, I1371V, has also been associated with astrocytic dysfunction in the glutathione machinery and its capability to mitigate oxidative stress [114]. Consistently, in microglial models, the loss of *LRRK2* was associated with changes in the inflammatory profile and increased Nrf2-mediated mitochondrial protection [115]. These findings imply that LRRK2 activity may inhibit Nrf2-driven bioenergetic protection in the microglia, although this interaction might differ in neuronal populations [115].

Other authors have focused on the interplay between Nrf2 and DJ-1, which protects Nrf2 from binding to its inhibitor KEAP1, thereby enhancing its antioxidative and anti-inflammatory—anti-NLR family pyrin domain containing 3 (NLRP3)—actions [116]. The absence of *PARK7* alters the gene expression (Figure 2) in a sex-specific manner in mouse midbrain astrocytes, which are the main cell type responsible for Nrf2 production, with the significant involvement of the Nrf2 and CYP1B1 regulatory axis, suggesting the higher sensitivity of males to the loss of DJ-1 [117]. Interestingly, the inhibition of glucocerebrosidase—encoded by the *GBA1* gene, mutations of which are also a risk factor for developing PD—selectively impaired the ability of female microglia to enhance the Nrf2-dependent detoxification pathway in neurons, attenuating the sex differences typically observed in this neuroprotective function [118]. These data could also be intriguing for the development of more targeted therapies involving this pathway.

Taken together, these studies provide evidence that PD-associated mutations disrupt Nrf2 signaling through several direct mechanisms: reduced nuclear translocation, transcriptional suppression, protein destabilization, and interference with Nrf2’s DNA-binding activity. These findings support the idea that Nrf2 dysfunction is not merely a downstream consequence of PD pathophysiology but rather a key contributor to the molecular cascade driving neurodegeneration in genetically defined PD.

In light of this knowledge, enhancing Nrf2 represents a clearly reasonable target in PD investigation. In this context, a couple of preclinical studies have evaluated melatonin’s potential as an inducer of Nrf2 to counteract the molecular pathology (mitochondrial dysfunction, oxidative stress, and protein aggregation) associated with PD (Figure 2). In zebrafish embryos exposed to the neurotoxin 1-methyl-4-phenyl-1,2,3,6-tetrahydropyridine (MPTP), melatonin restored the suppressed expression of the parkin/PINK1/DJ-1/MUL1 mitophagy network and improved mitochondrial function, suggesting a protective role in these relevant pathways [119]. Another study using the same model found that melatonin recovered previously disrupted circadian rhythms, motor activity patterns, and mitochondrial imbalances, helping to reestablish clock gene dynamics and neurobehavioral stability [120]. Furthermore, knockdown studies of *PINK1* in zebrafish showed mitochondrial defects and increased ROS levels that were mitigated by antioxidant treatments [121]. These results suggest that, in situations where the basal function of the protein was compromised, melatonin could contribute to the recovery of the correct dynamics.

Melatonin is also being studied in models of ferroptosis, a type of cell death characterized by the accumulation of lipid peroxidation, decreased glutathione levels, and glutathione peroxidase 4 (GPX4) activity, as a result of iron accumulation. Some researchers have reported melatonin’s important role in this process by activating multiple protective pathways, including sirtuin 6 (SIRT6), Nrf2 and ARE, HO-1, GPX4, MAPK kinases, PI3K/AKT/mTOR (mammalian Target of Rapamycin), and ferroportin [122]. In α-synuclein preformed fibril-induced PD mouse models, melatonin signaling through the overexpression of the melatonin 1 receptor (MT1) prevented ferroptotic cell death by preserving the sirtuin 1/Nrf2/HO-1/GPX4 antioxidant axis and reducing the iron load and α-synuclein aggregation, unlike MT1 knockdown and knockout, which worsened neuronal loss [123]. Other studies revealed that melatonin attenuated MPTP-induced autophagic dysregulation and α-synuclein buildup through a cyclin-dependent kinase 5 (CDK5)-dependent mechanism, suggesting an important role in modulating proteostasis under oxidative stress [124].

From a broader approach to PD-related mutations, in rodent models, melatonin reduced dopaminergic cell loss caused by the lentiviral delivery of the A30P mutant form of *SNCA*, implicating direct protective effects against familial point mutations [125]. Recently, melatonin was directly related with rescued Nrf2 levels in both cellular and mice models of D-gal-induced neuronal aging [126]. In line with these findings, due to Nrf2’s key role in the control of oxidative homeostasis, more studies on PD and melatonin induction have been conducted in recent years. Researchers have also developed nanoparticles that release melatonin in a controlled manner, promoting the selective removal of damaged mitochondria through mitophagy [127]. In vitro and in vivo studies demonstrated that these nanoparticles protect dopaminergic neurons, reduce α-synuclein protein accumulation, and improve motor function in PD models [127].

Several investigations using rotenone- and MPTP-induced PD rodent models confirmed that melatonin prevents mitochondrial damage and suppresses neuroinflammation [128,129]. More recently, in an MPP^+^ toxin-induced neuroblastoma model, it was reported that melatonin’s direct antioxidative mechanism led to a decrease in lipid hydroperoxide and 8-OhdG levels. Moreover, via the melatonin receptor, there was an activation of the melatonin receptor (Mel-R)/PI3K/Akt/Nrf2 antioxidative cascade [130].

Likewise, some melatonin derivatives have been shown to exert Nrf2 induction mechanisms in a pro-inflammatory glial context [131], under generally oxidative conditions [132], and in chemically induced PD cell models [133].

Collectively, these studies suggest that melatonin confers protection against neurodegeneration in PD via multiple mechanisms, including Nrf2 activation, but primarily in *PINK1* and *SNCA* models. Nevertheless, direct evidence involving other familial mutations—such as *LRRK2* and other *PARK* genes—although promising [134], remains limited. Melatonin is also being evaluated as an adjunctive treatment in PD because of many of its properties. Meta-analyses of clinical trials indicate that it has significant potential to improve disease symptoms, especially in relation to sleep disturbances and patients’ quality of life. Therefore, further study of the pathways involved—such as Nrf2—holds great potential for the development of more specific therapies in the future.

## 4. Concluding Remarks and Future Perspectives

The genetic mutations underpinning PD and FTD have provided critical insights into the molecular mechanisms driving neurodegeneration. Mutations in genes—such as *LRRK2*, *SNCA*, *PINK1*, *DJ-1*, and *MAPT*—disrupt fundamental cellular processes including mitochondrial function, protein homeostasis, and oxidative stress regulation. These dysfunctions converge on a shared pathological hallmark—elevated oxidative damage and impaired cellular antioxidant defenses. Nonetheless, Nrf2 dysfunction is emerging not only as a downstream consequence of PD but also as a key contributor to its pathogenesis, particularly in these familial forms. In a parallel manner, in tauopathies, an inverse correlation between *MAPT* and Nrf2 expression suggests that Nrf2 suppression may actively contribute to tau-driven neurodegeneration, further highlighting its central role across different proteinopathies. Within this context, the neuroprotective actions of Nrf2 and melatonin emerge as key modulators capable of counteracting mutation-induced neurotoxicity.

The Nrf2 pathway serves as a master regulator of cellular antioxidant responses, orchestrating the expression of a broad spectrum of detoxifying enzymes and cytoprotective proteins. Meanwhile, melatonin—beyond its classical role in circadian rhythm regulation—exhibits potent antioxidant, anti-inflammatory, and mitochondrial protective properties. The interaction between melatonin signaling and Nrf2 activation pathways presents a promising axis for therapeutic intervention; yet, the effects of specific genetic mutations on this interaction remain insufficiently studied.

Indeed, there are some limitations that should be taken into consideration in the context of therapeutic applications. Firstly, as mentioned, the effects of specific mutations on the melatonin-Nrf2 axis are still unknown. Therefore, the limited knowledge about the interaction between specific genetic mutations and the Nrf2 activation pathway entails a translational gap to human clinical application, as there is a lack of human clinical evidence in this context. Moreover, the mutation coverage in the literature is uneven, since most of the reviewed studies have focused on the interaction between Nrf2 and specific *MAPT* mutations, particularly *P301S* and *P301L*, which alter the biochemical properties of the 4R tau isoform. However, the mechanisms by which other *MAPT* mutations influence Nrf2 signaling remain poorly understood. Expanding the research to encompass a broader range of *MAPT* variants could help determine whether Nrf2 dysregulation represents a common pathological mechanism across familial tauopathies and may support the development of mutation-specific interventions. In contrast, within the context of PD, Nrf2 dysregulation has been widely demonstrated across various experimental models involving several of the previously discussed proteins. Yet, in more mutation-specific studies, attention has been directed primarily toward *PINK* or *SNCA*, given their higher prevalence. Consequently, information on mutations in other genes such as *LRRK2*, *DJ-1*, *VPS35*, or *GBA1* is still limited. Emerging efforts are now investigating how additional genetic alterations—such as mutations in *SNCA*—impact Nrf2 signaling, a topic of growing interest.

Concurrently, the potential of melatonin to activate the Nrf2 pathway in models harboring *MAPT* mutations underscores its promise as a therapeutic approach for familial tauopathies, particularly FTD. Further investigation is necessary in order to optimize the treatment parameters and validate its efficacy. Additionally, exploring whether tauopathy-linked mutations affect melatonin receptor functionality could reveal novel therapeutic targets for FTD. As for PD, melatonin’s neuroprotective effects are better established, with its actions increasingly linked to molecular pathways involving Nrf2. Nonetheless, efforts regarding the role of this molecule in PD are in the earlier stages, such as understanding the effects on the general symptoms associated with the disease. The current literature seeks to clarify the mechanisms and cellular pathways through which it may have beneficial effects. A broader approach than point-mutation models is currently being used. Therefore, the modulation of Nrf2 by melatonin in different PD mutations remains largely unexplored, since much is still unknown about the complexity and specificity of these interactions.

A more comprehensive understanding of how different PD-related mutations modulate Nrf2 and melatonin signaling may illuminate critical nodes within the disease-modifying network. To date, the number of clinical studies involving melatonin directly administered to patients with AD, PD, or FTD is very scarce. To our knowledge, only a few small clinical trials have been conducted, focusing mainly on sleep disturbances, both in PD [135] and AD [136], which show that oral melatonin reduces insomnia and improves sleep quality in patients. However, disease-modifying evidence via Nrf2 is quite limited. There is no consensus regarding the doses used, although most of those reported are below 10 mg/kg, nor is there any information on whether other non-oral routes of administration might be more effective. Other Nrf2 activators such as dimethyl fumarate [137] and omaveloxolone [138] are already in use as treatments for other neurodegenerative diseases, highlighting the potential of melatonin in this context. Ultimately, deepening our grasp of the balance between pathology and protection could mark a turning point in designing treatments that genuinely improve patient outcomes. Advancing this knowledge through mechanistic studies focused on specific mutations and genetically stratified clinical trials will be essential for developing targeted precision therapies.
